# Definitions and roles of a skilled birth attendant: a mapping exercise from four South-Asian countries

**DOI:** 10.1111/aogs.12166

**Published:** 2013-06-15

**Authors:** Bettina Utz, Ghazna Siddiqui, Adetoro Adegoke, Nynke Van Den Broek

**Affiliations:** Maternal and Newborn Health Unit, Liverpool School of Tropical MedicineLiverpool, UK

**Keywords:** Skilled birth attendant, emergency obstetric care, signal functions, maternal mortality, South Asia

## Abstract

*Objective.* To identify which cadres of healthcare providers are considered to be skilled birth attendants in South Asia, which of the signal functions of emergency obstetric care each cadre is reported to provide and whether this is included in their training and legislation. *Design.* Cross-sectional, descriptive study. *Setting.* Bangladesh, India, Nepal and Pakistan. *Sample.* Thirty-three key informants involved in training, regulation, recruitment and deployment of healthcare providers. *Methods.* Between November 2011 and March 2012, structured questionnaires were sent out to key informants by email followed up by face-to-face or telephone interviews. *Main outcome measures.* Mapping of definitions and roles of healthcare providers in four South Asian countries to assess which cadres are skilled birth attendants. *Results.* Cadres of healthcare providers expected to provide skilled birth attendance differ across countries. Although most identified cadres administer parenteral antibiotics, oxytocics and perform newborn resuscitation; administration of anticonvulsants varies by country. Manual removal of the placenta, removal of retained products of conception and assisted vaginal delivery are not provided by all cadres expected to provide skilled birth attendance. *Conclusion.* Key signal functions of emergency obstetric care are often provided by medical doctors only. Provision of such potentially life-saving interventions by more healthcare provider cadres expected to function as skilled birth attendants can save lives. Ensuring better training and legislation are in place for this is crucial.

## Key Message

Enabling all healthcare providers who are expected to provide skilled birth attendance to provide the signal functions of emergency obstetric care is urgently needed.

## Introduction

At the turn of the century, the international community agreed upon eight Millennium Development Goals (MDG), two of which are specifically related to maternal and newborn health (MDG 4 and 5). The maternal mortality ratio and the proportion of births attended by a skilled birth attendant (SBA) are two indicators that are used to monitor progress towards the attainment of MDG 5 (1). A maternal death is a relatively rare event and trends in maternal mortality over time are difficult to measure. The “proportion of births attended by skilled health personnel” is therefore often used as a more appropriate proxy indicator to track progress towards MDG 5. Skilled attendance at birth requires two key components: an SBA and an enabling environment that includes drugs and equipment, a functional referral system and enabling policies (2–4). Studies have demonstrated a positive correlation between the proportion of deliveries taking place with an SBA and a reduction in maternal deaths (5,6). The aim is to have a skilled attendant present at 90% of all births by 2015 (7). However, the World Health Organization estimates of coverage show that in 2011, only 18% of births in Bangladesh were attended by skilled personnel, in Nepal this was 19%, Pakistan 39% and India 47% (8).

An SBA is defined as “an accredited health professional such as a midwife, doctor or nurse who has been educated and trained to proficiency in the skills needed to manage normal (uncomplicated) pregnancies, childbirth and the immediate postnatal period, and in the identification, management and referral of complications in women and newborns” (3). Potentially life-threatening complications require immediate recognition and management, which is included in the package of care described internationally as emergency (or essential) obstetric care (EOC). Currently, EOC consists of nine interventions (or signal functions). Seven of these signal functions constitute what is referred to internationally as basic EOC. These include parenteral administration of antibiotics, oxytocics and anticonvulsants, manual removal of a retained placenta, removal of retained products of conception, assisted vaginal delivery (ventouse delivery) and newborn resuscitation using a bag and mask. Two additional signal functions – cesarean section and blood transfusion – constitute comprehensive EOC (9).

Currently, a multitude of different cadres of healthcare workers provide care during pregnancy and childbirth but it is not always clear if these providers are considered to be an SBA according to the existing international definition or which of the signal functions of EOC these healthcare providers can and are expected to perform (9,10). The objectives of this study were to explore which of the existing cadres of healthcare providers are considered SBAs in four countries in Asia, the length of their training, which signal functions of EOC each cadre of provider is expected to perform and whether training and legislation are in place to support this.

## Material and methods

Between November 2011 and March 2012, we carried out a cross-sectional, descriptive study in four South Asian countries: Bangladesh, India, Nepal and Pakistan. The countries were chosen purposively because of their involvement in the Making it Happen programme (11) which aims to build capacity for healthcare providers to provide skilled birth attendance and emergency obstetric and early newborn care. Data were collected using a structured questionnaire in English. Key informants were purposively selected according to their involvement in training, regulation, recruitment and deployment of SBAs in these countries. Key informants included personnel working in the maternal and newborn or reproductive and child health units of ministries of health, key staff of regulatory bodies such as nursing and medical councils or professional associations and academic staff of teaching institutions (medical and nursing schools) involved in training and deployment.

Using a semi-structured questionnaire and telephone or face-to-face in-depth interviews, we obtained information for each country regarding: the cadres of staff providing maternity services, whether these cadres conducted deliveries, whether they were considered to be SBAs, which of the EOC signal functions they were expected to provide, or were trained and/or legislated to provide. Informed consent was obtained in writing from all participants before completing the questionnaire or answering any study-related questions. Key informants were free to decline participation or withdraw at any time without given reason. All participants were asked to complete the questionnaire by email, which was supplemented by teleconferences or face-to-face interviews in-country. In the case of conflicting answers from key informants in the same country, all key informants were contacted again to clarify diverging information until consensus was reached. In cases where there was no consensus, the majority view is presented in this paper. Data were entered and analyzed using Microsoft Excel 2007.

## Results

A total of 33 questionnaires were completed. The overall response rate for the initial round of questionnaires sent out by email was 40.7% (33/81): 33.3% in India (7/21) and Nepal (5/15), 35% in Bangladesh (7/20) and 56% in Pakistan (14/25). Of the 33 questionnaires, 40.6% were completed by informants from regulatory bodies or professional associations (Nursing/Medical Boards), 40.6% by academic staff of teaching institutions and 18.8% by informants from Ministries of Health. The profile and distribution on non-respondents was similar to that of respondents.

The cadres of healthcare providers considered to be an SBA in each country are presented in Table 1. The number of cadres ranged from seven in Bangladesh and Nepal, eight in India to nine in Pakistan. In total across these countries, there were 13 cadres of providers considered to be SBA, of which six could be categorized as “medical” and seven as “nursing” cadres. Medical cadres such as obstetrician–gynecologists and medical officers with additional training in obstetrics as well as nursing cadres such as staff nurses and senior staff nurses are considered SBAs in all the countries. Job titles differed between countries and not all cadres were considered an SBA in all countries. The length of training for obstetrician–gynecologists and medical doctors/officers, staff nurses and senior staff nurses is comparable across the four countries. Major differences in length of training exist for three cadres: medical assistants, SBAs working at community level and lady health visitors. In India and Nepal, there is no cadre called community SBA; instead assistant nurse midwives are employed at community level and are trained for 18 months in both countries. Lady health visitors, a recognized cadre in both India and Pakistan, are considered SBA but the length of their training differs. Regulatory bodies (nursing or medical councils) exist for all cadres considered to be SBAs and formal registration is mandatory and in place for these cadres. No regulatory bodies were reported to exist for maternal and child health workers or for traditional birth attendants.

**Table:** Cadres of healthcare providers, whether considered a skilled birth attendant (SBA) or not, and training duration

Cadre	Bangladesh	India	Nepal	Pakistan
Obstetrician	5 years (MBBS) + 1 year for Diploma (DGO), 3 years MD to 4 years FCPS	5 years (MBBS) + 4 years specialist training	5 years (MBBS) + 3 years	5 years (MBBS) + 4 years specialist training
Medical Officer with additional training in O&G	5 years (MBBS) + 6 months or 1 year EOC training	5 years (MBBS) + 16 weeks EMOC or 18 weeks for anesthesia training	5 years (MBBS) + 6 months O&G training	5 years (MBBS) + 1 year
Medical Officer without additional training in O&G	5 years (MBBS)	5 years (MBBS)	5 years (MBBS)	5 years (MBBS)
MDGP	NA	NA	5 + 3 years (4–6 months O&G training)	NA
Ayurvedic Doctor	NA	5.5 years^1^	NA	NA
Medical Assistant	3 years^2^	NA	3 years	1 year
Sub-Assistant Community Medical Officer	3 years^2^	NA	NA	NA
Senior Staff Nurse	4 years previously; now 3 years	4 years	4 years	4 years
Staff Nurse	3 years	3 years	3 years	3 years
Assistant Staff Nurse	3 years^2^	NA	NA	NA
Midwife	No separate midwifery cadre yet; training to start 2012	No separate training, nurses with midwifery training are considered “midwives” (SSN)	No separate training, nurses with midwifery training are considered “midwives” (SSN)	18 months *Direct entry (community midwife) or via nursing (RNM)*
Auxiliary Nurse Midwife	NA	18 months (from 2012: 2 years)	18 months	NA
Family Welfare Visitor	18 months (not including Midwifery), some FWVs have additional 6 months SBA training	NA	NA	NA
Lady Health Visitor	NA	ANM training + additional 6 months training	NA	18 months
Family Health Worker	NA	NA	NA	12 months
Maternal and Child Health Worker	NA	NA	6 months^2^	NA
Community SBA	6 months	NA	NA	18 months
Traditional Birth Attendant	None	None	None	None

ANM, Auxiliary Nurse Midwife; DGO, Diploma Gynecology & Obstetrics; FCPS, Fellow of College of Physicians and Surgeons; MBBS, Bachelor of Medicine; MDGP, Medical Doctor with General Practice training; O&G, Obstetrics & Gynecology; RNM, Registered Nurse Midwife; SSN, Senior Staff Nurse; NA, cadre not present; 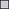

1 Considered as SBA only if trained (21-day SBA training).

2 Not conducting deliveries.

Key informants were asked to clarify which cadres of healthcare providers are expected to perform each of the signal functions of EOC and conformity between performance (P), training (T) and authorization (A) to perform each EOC signal function was examined (Table 2). Across all four countries the healthcare providers reported to be SBAs perform parenteral administration of antibiotics and oxytocics as well as newborn resuscitation with a bag and mask. Anticonvulsants are administered by 80–100% of SBA cadres in Pakistan, India and Nepal. In Bangladesh, anticonvulsants were given by only 43% of cadres expected to function as an SBA. Manual removal of a retained placenta, removal of retained products of conception and assisted vaginal delivery were mainly performed by specialist and general medical doctors; whereas, the majority of nursing cadres did not conduct these procedures. In some settings, healthcare providers are trained but in practice do not perform certain signal functions, such as in Nepal five of seven cadres are trained to perform manual removal of placenta, but only three are authorized to perform this. Conversely, some healthcare providers perform signal functions but this is not included in their training currently, such as in Bangladesh all seven cadres perform newborn resuscitation but this is included in training for only four.

**Table 2:** Performance (P), authorization (A) and training (T) of emergency obstetric care signal functions by cadres of healthcare providers considered to be skilled birth attendants (SBA)

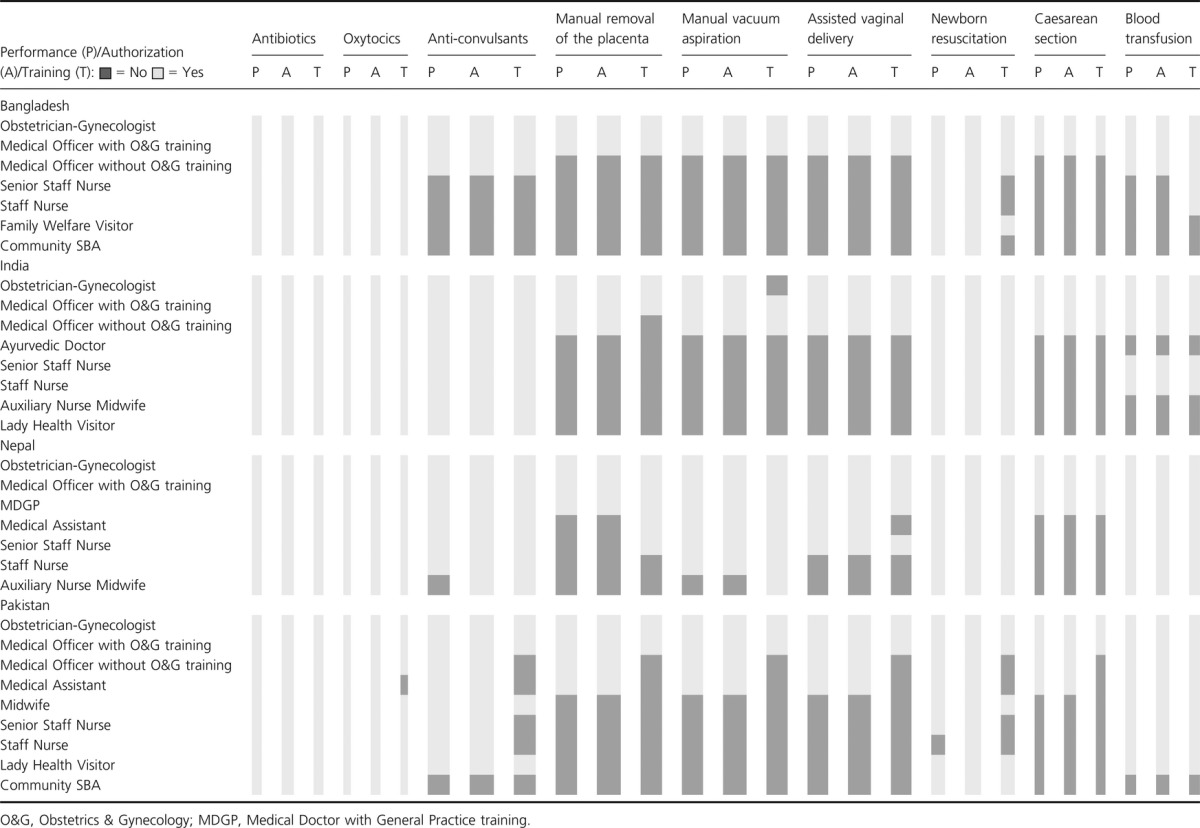

**Figure 1:**
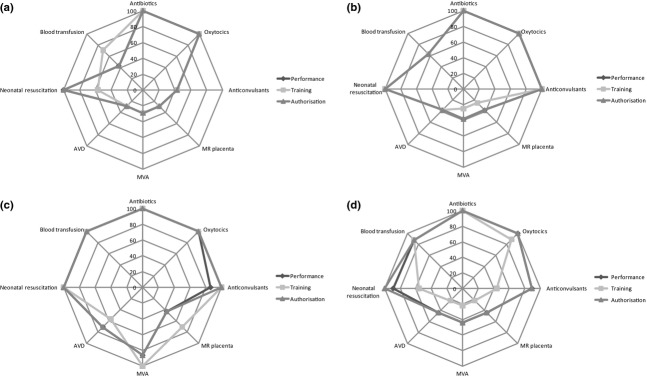
Proportion of healthcare provider cadres considered to be a skilled birth attendant reported to perform, trained and authorised to perform signal functions of emergency obstetric care in Bangladesh (a), India (b), Nepal (c) and Pakistan (d).

Figure 1 shows the percentage of healthcare providers considered to be SBAs who perform, are trained and authorized to perform the signal functions of EOC for each country.

## Discussion

Skilled birth attendance is essential to save maternal and newborn lives and key to attaining MDG 4 and 5. Ensuring that there are sufficient competent healthcare providers who can be considered SBAs is therefore crucial. This study mapped out the different cadres of health workers providing maternity services in these four countries in Asia and reports on whether these cadres are expected to provide skilled birth attendance, what they are trained and legislated to do and whether this includes the provision of EOC. This study shows that not all healthcare providers who currently conduct deliveries in South Asia are considered to be SBAs by key informants from ministries of health, regulatory bodies such as nursing and medical councils or professional associations.

For any healthcare provider who is expected to provide skilled birth attendance, training and legislation needs to be in place (12,13). This includes training and legislation regarding the signal functions of EOC. It is important that it is clear to the healthcare providers themselves, as well as to the staff supervising and managing the healthcare provider, which of the signal functions of EOC can be provided. This study shows that in a number of settings in South Asia this is currently not clear. Our findings are in line with a previous study from sub-Saharan Africa that also reported a lack of clarity and consensus with regard to which signal functions of EOC could be provided by different cadres of SBAs (10). Both this study and the previous one from sub-Saharan Africa report that key signal functions of EOC (manual removal of a retained placenta, removal of retained products of conception and assisted vaginal delivery) are currently mainly provided by medical staff.

Because of the global scarcity of healthcare providers, healthcare facilities often have no allocation of medical doctors and nursing or midwifery staff are expected to provide the majority of maternity care. It is, therefore, important that existing nursing and midwifery cadres are trained in the immediate recognition and management of obstetric complications and legislated to perform these. “Task shifting” is the term used in the literature to describe the additional allocation of tasks previously only provided by medical staff to healthcare providers who are nurses or midwives. This has been an effective strategy in other settings (14,15). For maternity care, task shifting means that providers who were previously not able to provide components of EOC will be enabled to do so. Providing additional “skills and drills” practical training has proved to be effective in increasing the knowledge and skills of healthcare providers (16). However, training on its own does not guarantee that healthcare providers apply the new knowledge and skills they have acquired. In India, medical officers received additional training in obstetrics. However, it was observed that only those doctors with an interest in obstetrics who were posted to facilities with a sufficiently high caseload were able to put acquired knowledge and skills into practice (17). This illustrates that even if training and legislation are in place, motivation and an enabling environment need to be present (12,13,18).

Similarly, research has shown that in some settings, trained and competent healthcare providers are in place but the staff lack the authorization from a professional body to provide components of EOC (2,3,10), constituting a poor use of existing staff (18). In the four South Asian countries included in this study, the different cadres of healthcare providers expected to provide skilled birth attendance were authorized (legislated) to provide most of the interventions they performed and it was rather lack of training that was highlighted as a reason why not all signal functions of EOC were provided. Review of the pre-service curricula of healthcare provider cadres expected to function as SBAs will clarify gaps in existing training (19). Recently the International Confederation of Midwives has provided guidelines to inform pre-service training curricula for cadres of staff providing skilled birth attendance (20). Assessment of healthcare providers’ competence in the work place will also help to determine if additional training is required.

Our mapping exercise revealed that obtaining consistent information from a country can be challenging. There is often a lack of clarity and agreement between different institutions and organizations responsible for training, legislation and employment of healthcare providers. Representatives of ministries of health, regulatory bodies and training institutions are often not aware of discrepancies that exist between performance, training and regulation. Therefore, there is a need to establish clear guidelines and disseminate any already existing guidelines describing the role and responsibility of each cadre of staff providing maternity services. This must include dissemination to all involved in monitoring, supervision, training and accreditation of healthcare providers.

A limitation of this study is that the information was provided by key informants working at the government, senior management and training institutions in each country who may not be aware of the exact reality on the ground. Although consensus among key informants was obtained, information provided was not verified by comparison with training curricula or direct observation of healthcare provider practice. There is a need for further research that would clarify what healthcare providers working in different types of health facilities are able to do. This would include direct observation and assessment of practice to establish which signal functions of EOC are performed (or not) and to identify the various barriers and enablers for this.

## Conclusion

Strategies encouraging delivery to take place with an SBA can only be successful if health workers have the necessary knowledge and skills to provide signal functions of EOC. The results of this study show that currently only a low percentage of healthcare providers who work as SBAs perform these key life-saving interventions in South Asia.

## Abbreviations

EOC: emergency obstetric care
MDG: Millennium Development Goals
SBA: skilled birth attendant
